# Bearing Fault Diagnosis of Induction Motors Using a Genetic Algorithm and Machine Learning Classifiers

**DOI:** 10.3390/s20071884

**Published:** 2020-03-28

**Authors:** Rafia Nishat Toma, Alexander E. Prosvirin, Jong-Myon Kim

**Affiliations:** School of Electrical, Electronics and Computer Engineering, University of Ulsan, Ulsan 44610, Korea; rafiatoma.eceku@gmail.com (R.N.T.); a.prosvirin@hotmail.com (A.E.P.)

**Keywords:** bearing fault diagnosis, condition monitoring, decision tree, genetic algorithm, induction motors, KNN

## Abstract

Efficient fault diagnosis of electrical and mechanical anomalies in induction motors (IMs) is challenging but necessary to ensure safety and economical operation in industries. Research has shown that bearing faults are the most frequently occurring faults in IMs. The vibration signals carry rich information about bearing health conditions and are commonly utilized for fault diagnosis in bearings. However, collecting these signals is expensive and sometimes impractical because it requires the use of external sensors. The external sensors demand enough space and are difficult to install in inaccessible sites. To overcome these disadvantages, motor current signal-based bearing fault diagnosis methods offer an attractive solution. As such, this paper proposes a hybrid motor-current data-driven approach that utilizes statistical features, genetic algorithm (GA) and machine learning models for bearing fault diagnosis. First, the statistical features are extracted from the motor current signals. Second, the GA is utilized to reduce the number of features and select the most important ones from the feature database. Finally, three different classification algorithms namely KNN, decision tree, and random forest, are trained and tested using these features in order to evaluate the bearing faults. This combination of techniques increases the accuracy and reduces the computational complexity. The experimental results show that the three classifiers achieve an accuracy of more than 97%. In addition, the evaluation parameters such as precision, F1-score, sensitivity, and specificity show better performance. Finally, to validate the efficiency of the proposed model, it is compared with some recently adopted techniques. The comparison results demonstrate that the suggested technique is promising for diagnosis of IM bearing faults.

## 1. Introduction

Induction motors, also known as asynchronous motors, are generally used as prime movers in the manufacturing, transportation, petrochemical, and power systems industries because of their rugged design, ease of control, low cost, high reliability, high overload capability, and efficiency. Due to their long operating times, the motors are subjected to many electrical and mechanical stresses, so the possibility of faults cannot be ignored. The cause of a failure can be inherent to the machine or caused by the operating conditions. The initial fault results in motor failure, which leads to downtime and increased production loss if it is not diagnosed in the initial stages. To avoid major losses, fault diagnosis in induction motors has recently received extensive attention among researchers [1,2]. To reduce significant damage, lengthen machine life, increase machinery accessibility, and reduce breakdown maintenance, effective fault diagnosis and condition monitoring is important. Fault detection helps to avoid unexpected interruptions as well as fatal damage of the whole drive system, whereas condition monitoring ensures decreased maintenance costs and increased reliability [3]. 

Any of the components in an induction motor can be damaged by overloading, abrasion, unbalanced loads, or electrical stress. Motor faults are categorized into four groups: bearing, stator, rotor faults, and other faults [4]. Of these, the possibility of incurring a bearing fault is the highest, which is more than 40% as discovered in research by General Electric Co. and IEEE-IGA [5], which indicates that bearing faults are the main cause of motor failures. This supports the fact that bearing fault diagnosis is important in the condition monitoring of rotating machinery. 

Machine-learning-based condition monitoring systems work by utilizing the historical data collected from the machine through various sensors under different working conditions. Feature extraction techniques are then applied to the raw signals to create a feature matrix, and finally the models are trained by these features. The sensor output signals are essentially time series signals, and features are generally extracted using different frequency domain, time domain, or time-frequency domain analysis techniques. The frequency domain analysis techniques include envelope analysis [6] and high-order spectral analysis [7]. Time domain analysis techniques comprise root mean square, high-order statistics, and the short impulse method [8,9]. Time-frequency analysis techniques include wavelet transform, short-time Fourier transform, and Hilbert-Hung transform [10,11]. A basic understanding of motor health can be determined from time-domain analysis and is easy to implement, whereas frequency domain techniques are able to distinguish a characteristic frequency of the fault from noise because they lack sensitivity to noise. Another advantage of using frequency domain signals is that no previous information is required to determine the signature features [12,13]. However, frequency domain analysis is not suitable for non-stationary signals, which is the most common type of signal. Time-frequency analysis is gaining in popularity as it performs well for both stationary and nonstationary signals [14].

Various techniques have been investigated for the detection and diagnosis of motor faults, such as vibration analysis [15,16,17], electromagnetic field monitoring [18], motor current signal analysis (MCSA), chemical analysis, infrared signal analysis, acoustic signal analysis [19], and partial discharge measurement [20]. Vibration signal analysis has been a consistently used approach, however, it requires the use of costly vibration sensors. In addition, the placement and installation of sensors in the precise positions to pick up the vibration signals is a challenge. Furthermore, the surroundings may induce noise which makes the sensor readings unreliable. 

Alternatively, MCSA has gained attention due to some advantages compared with vibration monitoring. MCSA uses the embedded current signal of the control unit of the motor, thus no additional sensors are needed, which results in low cost and a less complex system. By using the current signal, it is possible to remotely monitor a large number of motors from one place. Additionally, the faulty motor signals are quite unique and not easily affected by the surrounding working environment. Many studies have verified the trustworthiness of using MCSA to detect and diagnose motor faults. The stator current has recently been studied for use in fault diagnosis. In [21], a method is proposed using permanent magnet synchronous machines (PMSMs) based on the statistical analysis of the harmonics of the stator current. Bearing damage is evaluated using wavelet and short time Fourier transforms and the stator current. Single broken rotor bar (BRB) faults are effectively diagnosed using MCSA in [22,23,24]. Stator-current-based diagnosis is mostly used in machine learning methods for fault detection [25]. Both the stator current and voltage are used with machine learning approaches in [26].

Another powerful computational algorithm for fault detection is independent component analysis (ICA). The main advantage of this method is that the multivariate signal is divided into additive subcomponents by evaluating the mutual statistical independence of signals acquired from non-Gaussian sources [27,28,29]. Because statistically independent components are extracted, ICA captures important data structures and includes signal separation and feature extraction. Many researchers have used ICA for fault diagnosis in induction motors [12]. Widodo et al. [30] used a support vector machine for fault diagnosis with 10 features extracted from a total of 78 vibration signals using the ICA method. Interest in the use of artificial intelligence (AI) techniques in the process of fault diagnosis is growing. In the motor condition monitoring sector, the most frequently used AI technique is the artificial neural network (ANN) because of its excellent pattern recognition ability [31]. It can also represent non-linear models without any information regarding the actual structure and produces results within a very short time, providing promising results in motor fault diagnosis [32,33]. Some techniques have been merged with traditional approaches to fault diagnosis [34], including fuzzy logic (FL), envelope techniques [35], Bayesian classifiers, and deep learning [36]. When the machine learning approach is used for fault analysis, the selection of features is a pivotal part of the process as not all of the features extracted from the raw signal contribute significantly to fault classification and therefore can be discarded to reduce computational complexity. To both increase the accuracy and speed up operations, genetic algorithm (GA) is an effective technique to select the most appropriate features for the fault diagnosis model. 

There are several feature selection approaches in practice, but GA is gaining popularity. It is a heuristic algorithm for finding the best subset of features and is particularly suitable for high-dimensional datasets for which an exhaustive search is not feasible. It selects the most relevant and discriminative features from the set of the originally extracted features. GAs have largely been used in image classification, as in [37] and [38], where a GA is applied to find the best features for an apple leaf classification and breast cancer diagnosis, respectively. They are also used for optimal feature selection from fNIR signals [39] and in text classification [40]. The application of GA to fault classification is relatively new. In [41], this approach is used for fault diagnosis in spur gears from vibration signals. In the induction motor fault classification, principle component analysis (PCA) is commonly studied for use in feature selection and there are plans to apply GA and observe the outcome.

In this research, a motor current signal is used to classify bearing faults for three conditions: bearings with an inner race fault, with an outer race fault, and a healthy bearing. To reduce the data dimension, the set of statistical features was extracted from the raw time-series signals and GA was applied to determine the most significant features for the classification model. Finally, three widely used classification models were trained using the discriminative feature subsets selected by GA. The conclusive results and comparison indicate that the selected features converged quickly during the training state and therefore provided good performance in the classification models. 

This majorly focuses on the following aspects:

(i) Condition monitoring and fault classification of induction motors utilizing motor current signal analysis approach
(ii) Utilization of GA to select significant features from a collection of time-domain features and ensure acceptable results from the reduced feature set with the inclusive model.
(iii) This work investigates optimization parameters for GA as well as for the three different classifiers, k-nearest neighbor (KNN), decision trees (DT), and random forest (RF), to achieve good performance in comparison with other researches.

The rest of this paper is organized as follows: Section 2 presents materials and methods, including information about the experimental testbed and dataset, feature extraction and selection methods, classification techniques, and finally, the proposed method used in this study. Section 3 presents our results validating the proposed method and comparisons with existing methods. Finally, conclusions are provided in Section 4.

## 2. Materials and Methods

### 2.1. Bearing Fault Signatures

**Figure 1:**
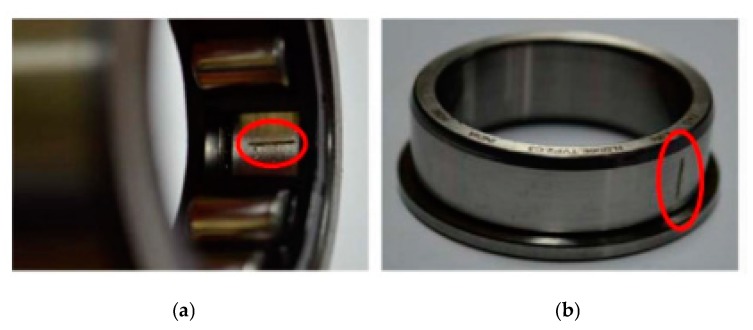
Bearing fault conditions. (**a**) bearing crack on the outer surface; (**b**) bearing crack on the inner surface.

Bearing faults are the most commonly occurring faults in IMs as shown in the previous section. Generally, rolling bearings are made up of an inner and an outer race which are separated by cylindrical rollers and balls. Damage like flaking and pitting can occur because of material fatigue or wearing [4] in any of these parts. In our analysis, we consider two bearing fault conditions, inner race and outer race fault, are illustrated in Figure 1. 

When a ball passes through a damaged point in a defective bearing, shock pulses having a characteristic frequency occur. The characteristic frequency can be calculated using the geometry of the rolling element and the rotational frequency, $f_{m}$. The fault causes vibrations that introduce anomalies into the air-gap flux density, causing an aberrance in the harmonics of the current signal. This change in the current signal indicates that bearing faults have occurred. The different characteristic frequencies for each fault mentioned in [4] are listed below.

Outer race defect:

(1) $$f_{outer=}\frac{N_{ball}}{2}.f_{m}.\left(1-\frac{D_{ball}}{D_{cage}}.cos\beta \right)$$

Inner race defect:

(2) $$f_{outer=}\frac{N_{ball}}{2}.f_{m}.\left(1-\frac{D_{ball}}{D_{cage}}.cos\beta \right)$$

Ball defect:

(3) $$f_{ball=}.f_{m}.\frac{D_{cage}}{D_{ball}}\left(1-\frac{D^{2}{}_{ball}}{D^{2}{}_{cage}}.cos^{2}\beta \right)$$

Cage defect:

(4) $$f_{cage=}\frac{1}{2}.f_{m}.\left(1-\frac{D_{ball}}{D_{cage}}.cos\beta \right)$$

where β is the contact angle of the balls, *Nball* is the number of balls or cylindrical rollers, *Dball* is the diameter of the ball, and *Dcage* is the cage diameter, which is also known as the roller or ball pitch diameter. The detail ball bearing geometry is described in [42].

A radial motion occurs between the stator and rotor due to bearing damage, which produces oscillations and induces characteristic fault frequencies into the current signals. The reason behind this is that the bearing defects cause radial displacement of the rotor relative to stator. It results in the fluctuations of the rotating eccentricity and load torque. This leads to variation in machine inductances causing amplitude, frequency and phase modulation of the motor-current signals. The motor current equation for a faulty bearing is provided in [43,44] as:

(5) $$i\left(t\right)=\overset{\infty }{\underset{k=1}{\sum }}i_{k}.\cos\left(\omega_{c_{k}}.t+\varphi \right)$$

where *φ* and $\omega_{c_{k}}$ denote the phase angle and angular velocity, respectively, and $\omega_{c_{k}}=\frac{2\pi f_{bearing}}{p}.$

Here, *f_bearing_* is the fault current harmonic frequency, and *p* denotes the pole pair number of the respective machine. Note that *f_bearing_* can be written as:

(6) $$f_{bearing}=\left|f_{s}\pm mf_{v}\right|$$

where *f_s_* is the supply or fundamental frequency, *m* = 1, 2, 3, … and indicates the harmonic indexes, and *f_v_* can be the inner race defect frequency, *f_inner_*, or the outer race defect frequency, *f_outer_*. The estimated fault signature frequencies can be calculated using the frequency auto search algorithm presented in [45]. Sometimes, the harmonics produced by bearing faults can be close to or overlap with noise frequencies and become difficult to distinguish, which creates problems in the detection of bearing faults [46]. Hence, when the bearing parameters are unknown and the inverter frequency changes, it becomes difficult to diagnose the bearing defects in an IM. 

### 2.2. Experimental Testbed and Dataset Acquisition

**Figure 2:**
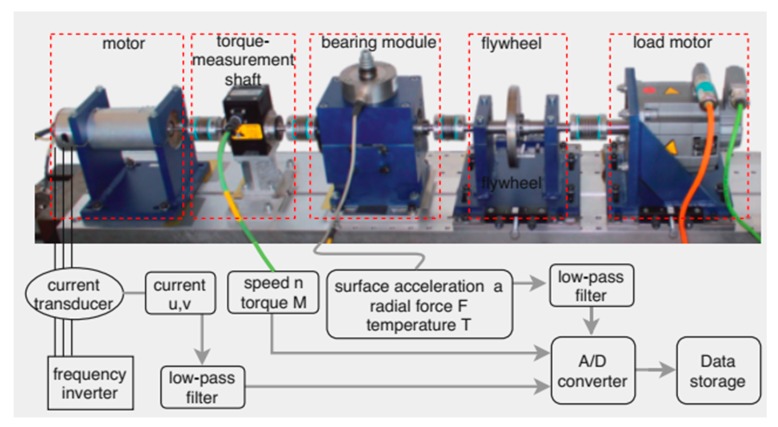
Schematic diagram of signal measurement.

The dataset used in this work is obtained from the Mechanical Engineering Construction and Drive Technology (KAt) Research datacenter of Paderborn University, Germany. The current signal dataset is produced from a test bed as shown in Figure 2, which consists of a 425 W permanent magnet synchronous motor controlled by a standard frequency inverter of switching frequency 16 kHz. The detailed experimental procedure is provided in [47]. Here, the test rig was operated under various working conditions to confirm its robustness as well as to investigate the impact of the operating parameters. To make the data more reliable, both real and artificially damaged bearings were used. Among the 32 different bearings used, six were healthy, 14 were naturally damaged, and in the remaining 12, the damage was created artificially. Regarding bearing damage categorization, the specific criteria were set by the researchers in [47] that allowed for grouping the bearings into four categories, among them the first three categories described the bearing properties, whereas the last category that was called “damage” was prepared according to ISO 15243. Furthermore, the appropriate directions of geometrical sizes of the cracks in bearings were assigned according to VD1 3832 (2013). Moreover, in the original study, the measured data was verified by means of envelope analysis and machine learning-based classification. 

The sets of bearings used in this dataset can be split into three groups based on their health state: healthy bearings, bearings with inner race faults, and bearings with an outer race fault. Four different operating conditions were created by varying the rotational speed, load torque, and radial force, as shown in Table 1. 

The motor phase currents were measured with a current transducer (LEM CKSR 15-NP) at two different phases denoted as Current Signal 1 (CS1) and Current Signal 2 (CS2). First, 20 measurements, each over a duration of 4 seconds, were taken for every bearing set presented in Table 2. Then, the collected signals were filtered using a 25 kHz low pass filter and finally sampled at a rate of 64 kHz. For each bearing set, 20 measurements were performed to determine the force, CS1, CS2, speed, torque, and vibration signal. Although the current signals from 32 bearing sets were recorded in the original dataset, the final dataset used in this study was constructed using the signals from 17 bearing sets picked from the three different health state classes. The bearing codes and types used in this research are provided in Table 2.

The final dataset can be expressed as a 136000 × 5118 matrix that contains two current signals from different phases (CS1 and CS2) and a label placed in the last column to differentiate the health states of the bearings.

**Figure 3:**
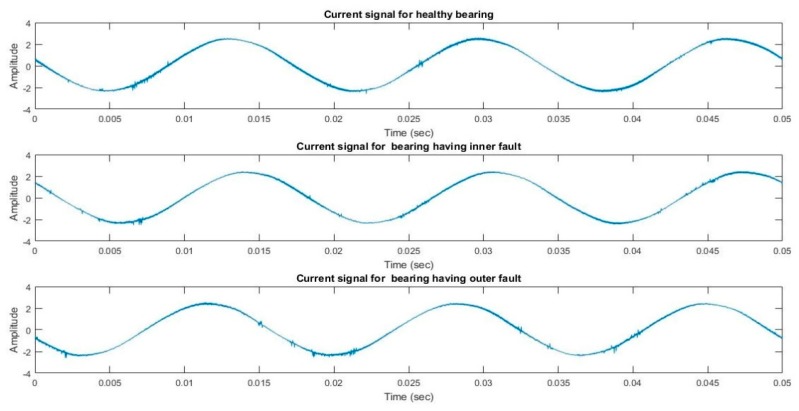
Motor current signal for three different conditions of bearing mentioned in Table 2.

The plot of current signals collected for three bearing states (healthy, inner fault, and outer fault) is provided in Figure 3. It can be seen that there are very subtle differences among the signals in time domain representation.

**Figure 4:**
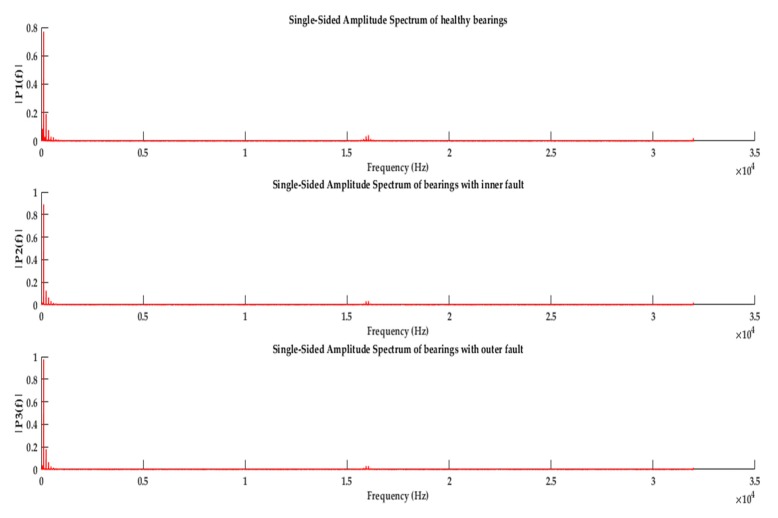
Frequency spectrum from MCS for healthy bearing, inner ring damage, and outer ring damage.

For demonstrating the bearing fault characteristics frequencies, the most common approach is to convert the current signal from time domain to frequency domain. The current signal is sampled at a rate of 64 kHz. After combining all the data, we performed the fast Fourier Transform (FFT) on the instances of three classes. To get better frequency resolution, the initial length of the FFT function is selected equal to the length of the data signal; however, for the representation purposes, the “mirrored” part of frequency spectrum has been removed. The obtained frequency responses are provided in Figure 4. Here, the characteristic frequencies are masked because of the external noise and presence of distributed damages. Therefore, with only characteristics frequency approaches, it is very difficult to detect fault. In such cases, denoising techniques and the machine learning models can be applied to classify different damages.

### 2.3. Feature Extraction

Feature extraction reduces the dimension of the initial raw data by converting it into smaller, more manageable groups for further processing. Most of the time, the raw dataset contains a high number of variables, thus requiring a large amount of computing resources to process. The main goal of this method is to select and convert the variables into features, effectively compacting the data which must be processed and precisely describing the original raw data set. Recently, on-line diagnosis for condition monitoring has gained attention because of its ability to detect incipient faults. However, a small amount of data is not enough for diagnosis, so directly measured signals are not adequate for on-line use. For effective fault diagnosis, a large data sample is required. Thus, to make the calculation easy, feature extraction becomes a crucial step that reserves critical information for final decision making.

Ten classical statistical feature parameters for condition assessment were computed from the time domain data. The time domain features extracted from the signal are mean, median, standard deviation, variance, sum, skewness, kurtosis, energy, root mean square, and crest factor. The mean is the average of the available data points and median is the number located at the middle when the data points are arranged in ascending order. Mean and median both are measures of central tendency, but median is not sensitive to outliers. Standard deviation and variance are the measures of variability. Variance is the average squared differences of the values in a data set from the mean. It indicates how far the data points are spread from the mean. Standard deviation is obtained by taking the square root of the variance. It is a statistic of dispersion of observations within a data set. The degree of distortion of a normal distribution is measured by skewness, which differentiates extreme values in one versus the other tail, which also represents lack of symmetry of a data set. However, kurtosis measures the existing outliers in the distribution. Finally, the crest factor represents the ratio of peak value to the effective value of any dataset. The mathematical equations for the described statistical features are listed in Table 3. Here, $x_{i}$, i =1, 2, …, N represents the motor current signal. *N, μ* and *σ* represent the number of data points, mean and standard deviation respectively. The time-domain features contain statistical details about the type of current signal, and because of their sensitivities, are good quality features [48]. 

### 2.4. Feature Selection with Genetic Algorithm

Feature selection is the process of detecting and eliminating irrelevant, less useful, and redundant features, and of discovering the most appropriate inputs for a classification model. A GA, proposed by J. H. Holland, uses a heuristic search that provides a satisfactory balance between computational complexity and optimal selection [49] and is effective for feature selection. It is a stochastic method for optimizing functions that is dependent on the mechanics of natural genetics and biological evolution and is able to discover accumulating information about a primarily unknown search space. It can be applied to high-dimensional data without any specific knowledge of the problem under study and can easily be parallelized in computer clusters. 

**Figure 5:**
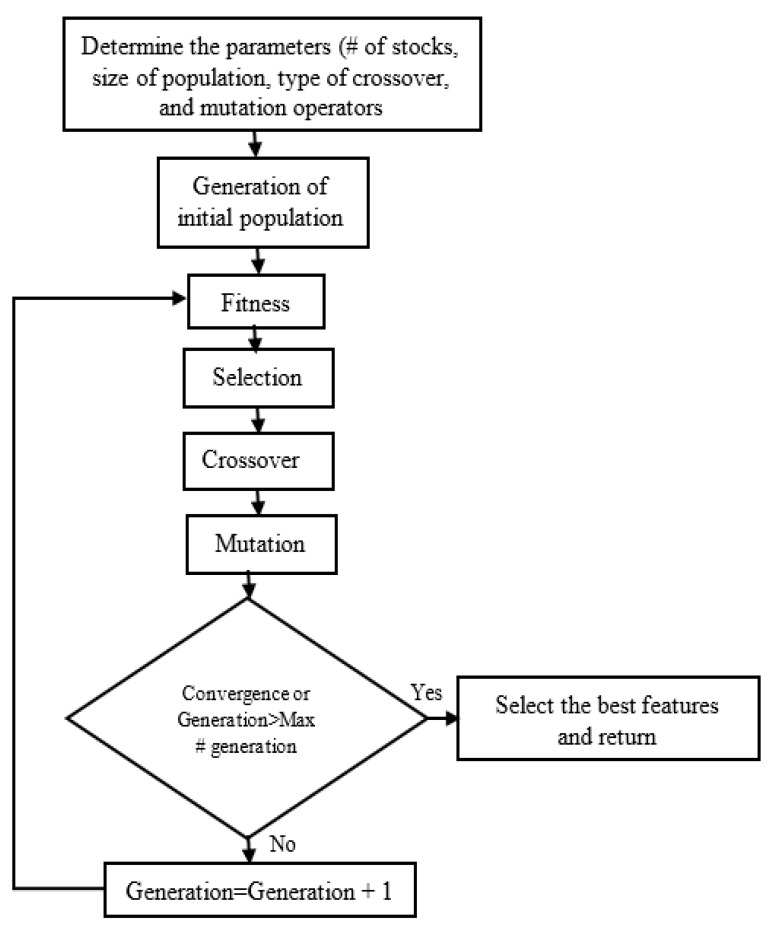
A flow chart of a genetic algorithm optimization.

The overall process of GA-based feature selection is provided in Figure 5. The initial population is created with the feature vector. Each member in the population is called a chromosome. The next crucial step is the selection of an appropriate evaluation function for successful application of GA to the problem domain. Each chromosome is assessed and categorized depending on how well it satisfies the objective function, and finally it is assigned to a probability of reproduction. In the selection process, the fittest individuals are selected to inherit the best characteristics. Several algorithms are applied in this process: c wheel selection, tournament selection, uniform selection, and remainder selection. Next, in the crossover stage, the next generation is formed by a simulated mating process and combines the best features of the parents. Once the children are generated in this crossover process, the mutation operation, which is a random modification of genes during reproduction, is applied to each child. This helps the children to inherit the best characteristics of their parents, and finally, a better solution to the problem will emerge. This process is then repeated with the new population until certain criteria are achieved. The algorithm ends when the population congregates at the optimal solution, or the highest number of generations is completed. 

### 2.5. K-nearest Neighbor (KNN)

After feature selection using GA, three different classifier algorithms are applied to classify the fault signature. To begin with, K-nearest neighbor (KNN) was chosen. This is a widely-used parametric classifier because of its simple architecture and low computational complexity [50]. It can also handle data with various characteristics, including nonlinear, multimodal, and non-Gaussian data. In the KNN classification algorithm, the position of the training samples is kept fixed, then for a new data sample, the distance between the training samples and the query data is measured. Using these distances, K samples are chosen from the training samples having the minimum distances. A distance metric, *d*, and a positive integer, K, act as decision making variables. The test data are assigned to the class with more samples in that region [51]. During the training process, the data remains in memory and for data with a very high dimension, the computational complexity is high. To reduce this complexity, we select the most informative and optimal features with a GA-based approach. 

**Figure 6:**
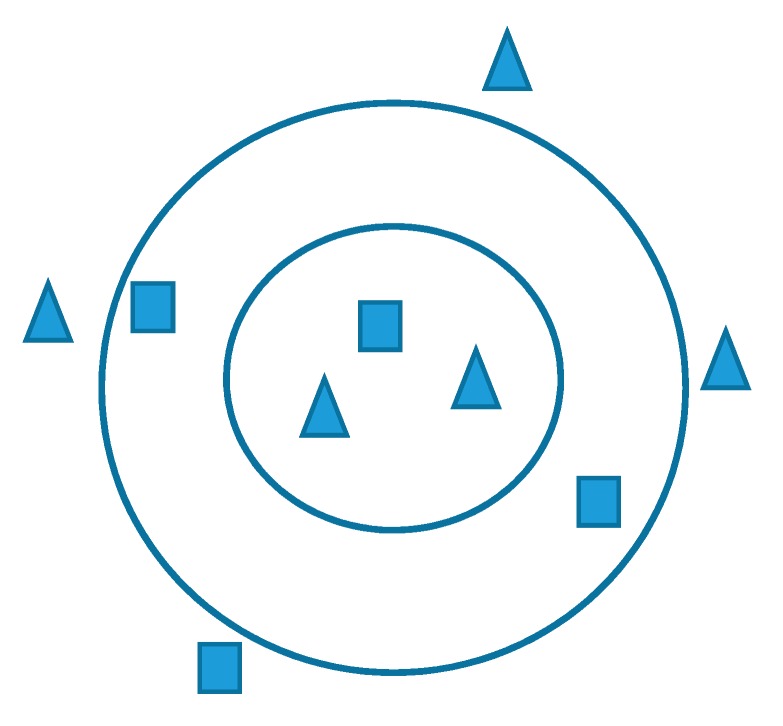
KNN algorithm for a situation with two classes and two features.

For example, consider two or three features in a classification process. To find a result, draw a circle centered on the input location and take the radius containing K sample values. Then, the class having more sample values within that circle is the result. Figure 6 shows KNN in detail. Here, for K = 3, inside the small circle, there exist one square and two triangles, so the new sample will belong to the triangle class. Similarly, for K = 5 and K = 9, the result will be square and triangle, respectively. The optimum value of K is crucial in defining the number of neighbors and the distance matric. In this work, a wide range of K values is examined to determine the optimal value for the training samples.

The Euclidean distance matric is used to compute the distance matric, which is a simple way to implement this method in a multidimensional input space and provides better comparative results than other erudite machine learning methods. The Euclidean distance between two points, *p* and *q*, can be calculated with the equation given below.

(17) $$d_{E}=\sqrt{\overset{n}{\underset{i=1}{\sum }}{\left(p_{i}-q_{i}\right)}^{2}}$$

### 2.6. Decision Tree

**Figure 7:**
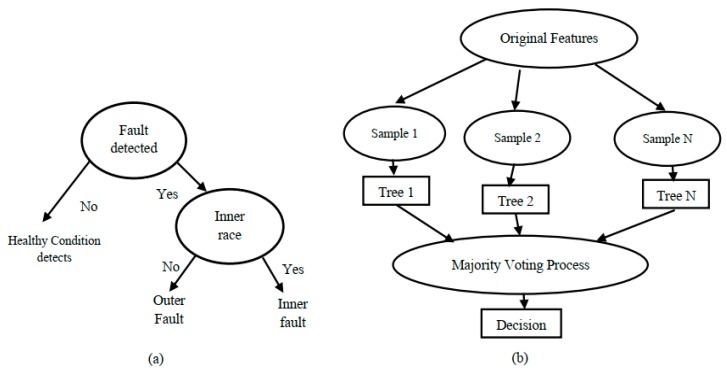
(**a**) Decision tree; (**b**) random forest architecture.

Among the various supervised learning models, some of the best and most-used methods are the tree-based techniques. They work well on both linear and non-linear systems because of their good stability and ease of interpretation, and most importantly, they allow for predictive modeling with high accuracy. A DT can be described as a graph that represents choices and their results in the form of a tree. This model consists of splitting processes, which divide a node into two or more sub-nodes, and pruning processes, in which the system removes sub-nodes of a decision node, which is illustrated in Figure 7a.

### 2.7. Random Forest

Random decision forests are one type of collective learning for both regression and classification. This technique works by constructing a multitude of decision trees during the training period, then defining the class which is the mode of the classification classes and predicting the mean value for regression of the individual tree. The advantage of RF over a decision tree is that it can overcome the overfitting problem. The number of trees, maximum depth, number of features in every split, and number of sample leaves are the significant parameters in RF. In most cases, the high number of trees helps to provide stable results for accurate calculation. Generally, the number of features controls the diversity of a tree in a forest such that a small number of features results in uncorrelated trees and a large value represents correlated trees. Therefore, a large number of trees can improve performance, but also increases the size and computational cost. In this work, a range of values for the above-mentioned parameters are tested. The resulting diagram of random forest algorithm is provided in Figure 7b.

### 2.8. Proposed Method 

In this work, motor current signals from two different phases under different operating conditions with the bearing sets of three health states are taken to form a large dataset. Then, 10 statistical features are extracted for each phase to build a feature matrix of 13600 × 20.

**Figure 8:**
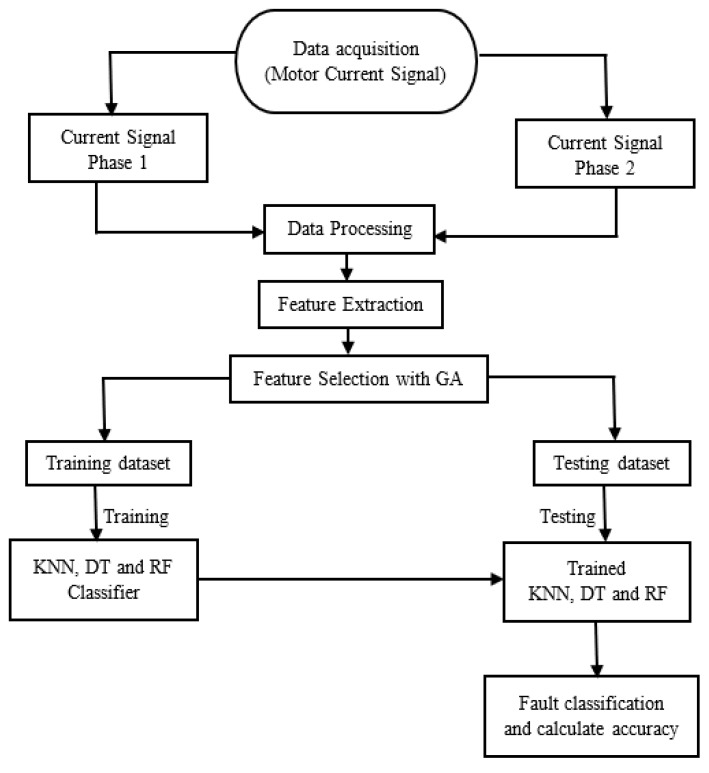
Schematic diagram of the proposed methodology.

GA is applied to select the discriminative features from this matrix to reduce its dimensions, which results in less computational complexity. Deciding the optimal number of features is crucial, as too many features may result in too much noise and with too few features, important information may be lost [20]. In this study, from the 20 extracted features, the GA selected a series of features (5, 6, 10, 11, 12, 14, 16, and 18) which were combined to make different input subsets. The classification loss of KNN algorithm (K = 3) is used for constructing the fitness function. This results in the most suitable input based on the predicted accuracy of the classification models. Finally, these discriminative features selected by the proposed GA framework are used as the inputs to three different machine learning classification algorithms to perform the fault classification task. In this study, the feature matrix obtained after feature selection process is randomly split into the training and testing subsets in 70:30 ratio for training and validating the fault diagnosis capabilities of the classification algorithms. Specifically, 9520 data instances are utilized for training the machine learning algorithms, whereas the remaining previously unseen by the trained classifiers 4080 samples are applied for validating the final fault classification performance. To ensure consistency in the results, the training was repeated several times and almost the same test accuracy was achieved for each iteration. The schematic diagram of the proposed method is given in Figure 8. 

### 2.9. Model Evaluation

The feature set selected using GA is used as the input for the three different classifier algorithms discussed in the previous section. Of the different evaluation parameters, the confusion matrix, overall accuracy, sensitivity, specificity, prediction, and the F1-score are considered as the performance metrics in this work. The evaluation parameters are calculated by the equations provided in Table 4.

Here, sensitivity indicates how appropriately the model is detecting events in the positive class, whereas specificity measures how precise is the assignment to the positive class. Most of the time, sensitivity and precision are reported pairwise because type I errors are calculated by sensitivity and type II errors by precision. Finally, the F-measure is the harmonic mean of sensitivity and precision.

## 3. Results and Discussion

In this section, the impacts of various GA parameters are discussed, then the performance of the classifier models is presented. To select the optimum values for the parameters of GA such as population size, mutation, and crossover, we have run the GA procedure for 100 generations.

**Figure 9:**
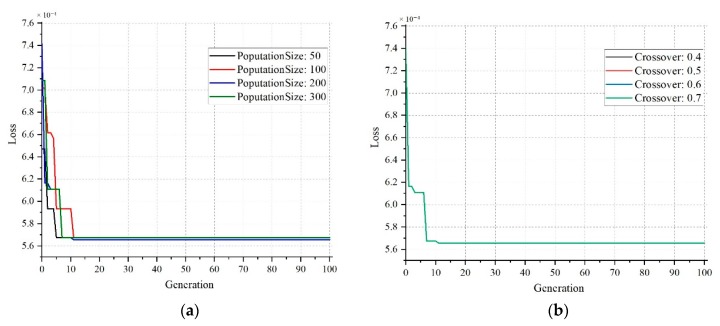
Effect of varying the population size (**a**) and crossover probability (**b**).

Figure 9a indicates that if a population size of 200 is chosen, the loss will be at minimum level of just below 5.7. For other population sizes, the loss is slightly higher than the minimum. Figure 9b shows that the loss curves follow the same trend regardless of the variations in the crossover probability. However, following the general practice of assigning the crossover probability within the range of 0.65 to 0.85, the crossover probability has been chosen to be 0.7 in this study.

**Figure 10:**
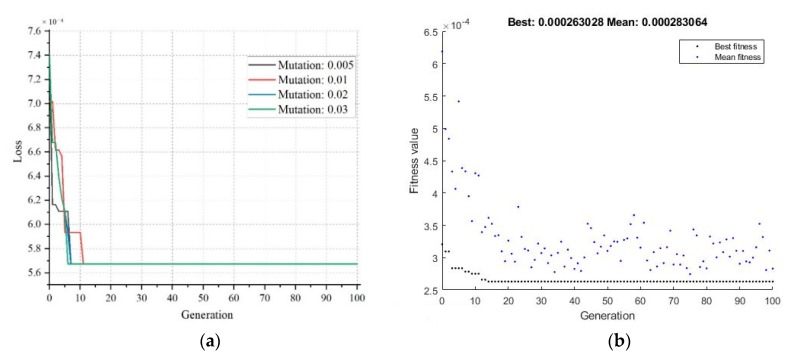
Effect of varying the mutation probability for estimating loss (**a**) and generations for fitness value (**b**).

A similar pattern can be observed for the mutation probability as shown in Figure 10a. It can be observed that after a certain number of generations, the values of loss curves become identical for different values of mutation probability. In this work, 0.03 was selected as the mutation probability value. For the fitness value estimation, the mean values began to converge as the generations increased, whereas the best fitness value remained the same at 2.6 × 10^-4^ after 15 generations. The list of finalized GA parameters is tabulated in Table 5. 

**Figure 11:**
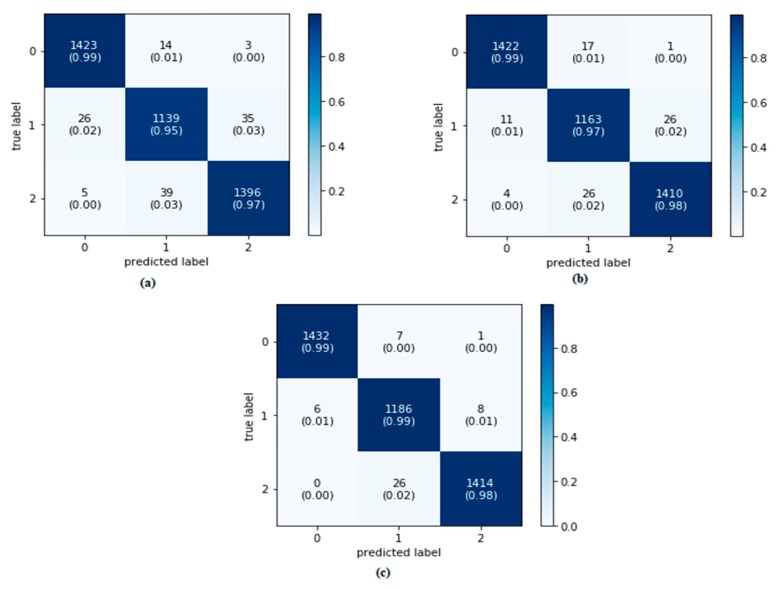
Confusion matrix for (**a**) KNN, (**b**) decision tree, and (**c**) random forest classifiers.

**Figure 12:**
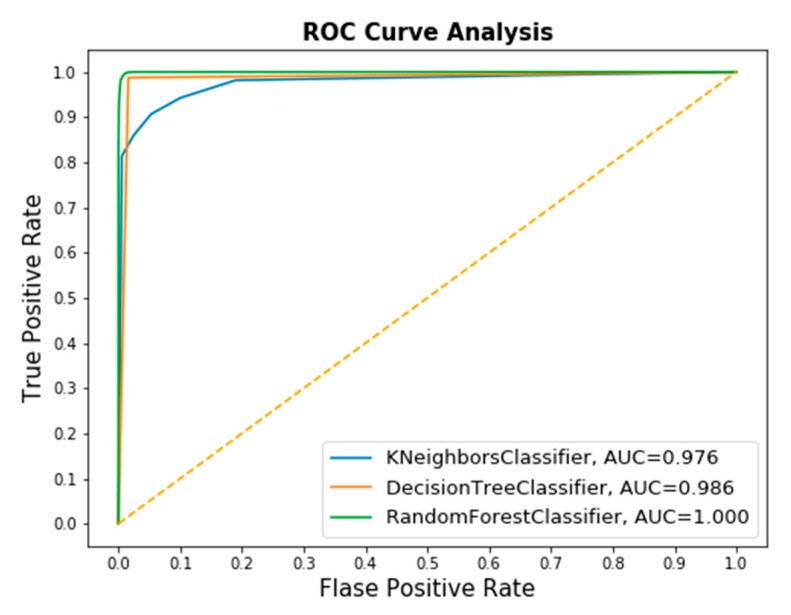
Combined ROC curve for the KNN, decision tree, and random forest classifiers.

The classifier’s performance was assessed using the confusion matrix and receiver operation characteristic (ROC) curve presented in Figure 11 and Figure 12, respectively. The evaluation parameters shown in Equations (18)–(22) were calculated from this confusion matrix. In ROC, the area under curve (AUC) was used because it provides a perception of how accurately a classification model performs on the dataset. The range of the AUC curve was from 0.5 to 1, where 0.5 represents a classifier that has not performed better than random guessing, and 1 indicates a good model with no misclassifications. The nearer the value is to 1, the better the model.

From the values we get from the confusion matrix, it can be said that the RF preforms better (99%) than that of KNN and DT to predict the true class. In addition, the AUC score for DT and RF are almost 1, which indicates that the model performs good in comparison with the KNN classifier. 

Table 6 lists the performance parameters. It can be concluded that all three classifier models performed well when trained with the features selected by GA, and the accuracy values were more than 97% for the applied classifiers. However, with RF classifier, more than 99.5% accuracy can be achieved.

To verify whether the application of two-phase current signals in conjunction with the proposed GA framework for selecting discriminative features benefits the fault diagnosis procedure, we also consider the experiment where only a one-phase current signal with the same statistical features is used as the input to machine learning classifiers. The comparison between the two-phase signal utilization with GA-based feature selection and the one-phase signal used with the same statistical feature parameters is demonstrated in Table 7.

As can be seen from this table, when only 10 statistical feature parameters extracted from a one- phase current signal are used for diagnosing bearing faults, the classification accuracy achieved by KNN, DT, and RF classifiers degrades in comparison with the proposed approach and is equal to 89.7%, 91.03%, and 91.1%, respectively. From this result, we can conclude that the proposed feature model built using the two--phase signal demonstrates better fault classification performance than the one where only a one-phase signal is utilized for feature extraction.

A comparison of the proposed method with other works is presented in Table 8. In [52], information fusion (IF) and deep learning techniques were applied on the same current signal dataset used in this work and achieved an accuracy of 98.3% with Multilayer Perceptron (MLP). C. Lessmeier et al. [47] employed wavelet packet decomposition (WPD) for feature extraction and then applied ensemble learning on the motor current signal and achieved an accuracy of 86.03%. A similar type of research conducted in [53] has proposed a fault detection method for induction motors utilizing empirical wavelet transform and CNN achieved 97.37% accuracy, which is also comparable with our result. In [54], authors used a vibration signal and CNN for fault detection and diagnosis, achieving accuracy between 88–99% for different ratios of data. Another method proposed in [55] attained 98% and 100% accuracy for detecting rotor fault and bearing fault respectively when using a CNN. In comparison with these recent studies, our proposed method produced a comparable result using only statistical features and GA for feature extraction and selection leading to less computational complexity than the other methods. 

## 4. Conclusions

In this paper, a motor-current data-driven approach based on a combination of statistical features, GA, and machine learning techniques is proposed for IM bearing fault diagnosis. To investigate the effective features, 20 statistical features are extracted using the motor current signal as the original input. Eight features are selected using GA, which are used to train three different classifiers for fault diagnosis. For feature selection, the most suitable genetic parameters for GA are selected, and a series of simulations is conducted to find the best accuracy using the classification models. The main goal is to reduce redundant data and the dimension of the original data set, resulting in less power and computational complexity for the models. The proposed system was tested using only the current signals from three different motor states. One condition is the healthy state, and the other conditions include two different bearing faults (inner race and outer race). The overall accuracy of fault classification is satisfactory, showing that the proposed method is promising for real time application. Additionally, to authenticate the efficacy of this model, a comparison with some recent work on the same dataset and several different datasets was presented, and the comparison indicates that the proposed method, employing only simple statistical features has comparable accuracy while requiring less computational complexity than other methods. If the vibration signal is considered, accuracy obtained by state-of-the-art CNN architecture is also comparable with the results obtained in this work where current signal is used. This indicates that current signal collected from a sensor less system is as effective as the conventional sensor-based system. In our future research work, we will investigate the applicability of frequency-domain analysis for benefiting the proposed approach for diagnosing rolling element bearings using the IM current signals. Furthermore, we will validate the applicability of the proposed methodology on other datasets where the diagnosis of mechanical faults using the current signals is considered.

## Figures and Tables

**Table 1:** Operating Conditions.

No	Rotational Speed (S) [rpm]	Load Torque (M) [Nm]	Radial Force (F) [N]
1	1500	0.7	1000
2	900	0.7	1000
3	1500	0.1	1000
4	1500	0.7	400

**Table 2:** Bearing sets for experiments.

Type of Bearing		Bearing Code	Label
**Healthy Bearing Data**		K001	0
		K002	
		K003	
		K004	
		K005	
		K006	
**Naturally Damaged Bearing Data**	**Outer Ring**	KA04	1
		KA15	
		KA16	
		KA22	
		KA30	
	**Inner Ring**	KI04	2
		KI14	
		KI16	
		KI17	
		KI18	
		KI21	

**Table 3:** Extracted statistical features from time domains for the feature matrix (x is the current signal).

Statistical Feature	Equation		Statistical Feature	Equation	
**Mean:**	$\mu =\frac{1}{N}\sum_{i-1}^{N}x_{i}$	(7)	**Skewness:**	$x_{skewness}=\frac{1}{N}\frac{\sum_{i=1}^{N}{\left(x_{i}-\mu \right)}^{3}}{\sigma^{3}}$	(8)
**Median:**	$median={\left(\frac{\left(N+1\right)}{2}\right)}^{th}value$	(9)	**Kurtosis:**	$x_{kurtosis}=\frac{1}{N}\frac{\sum_{i=1}^{N}{\left(x_{i}-\mu \right)}^{4}}{\sigma^{4}}$	(10)
**Standard Deviation:**	$\sigma =\sqrt{\frac{\sum_{i=1}^{N}{\left(x_{i}-\mu \right)}^{2}}{N-1}}$	(11)	**Energy:**	E = $\sum_{i=1}^{N}x_{i}{}^{2}$	(12)
**Variance:**	$\sigma^{2}=\frac{\sum_{i=1}^{N}{\left(x_{i}-\mu \right)}^{2}}{N-1}$	(13)	**RMS:**	$X_{rms}=\frac{\sqrt{\sum_{i=1}^{N}x_{i}{}^{2}}}{N}$	(14)
**Sum:**	$\mathrm{Sum}=\sum_{i-1}^{N}x_{i}$	(15)	**Crest factor:**	$C_{f}=\frac{X_{\max}}{X_{\min}}$	(16)

**Table 4:** Performance evaluation parameters.

$\mathrm{Precision}=\frac{\mathrm{TP}}{\mathrm{TP}+\mathrm{FP}}.$	(18)	$\mathrm{Sensitivity}=\frac{\mathrm{TP}}{\mathrm{TP}+\mathrm{FN}}$	(19)
$\;\mathrm{Specificity}=\frac{\mathrm{TN}}{\mathrm{FP}+\mathrm{TN}}$	(20)	$\;F1=2\times \frac{\mathrm{Precision}\times \mathrm{Sensitivity}}{\mathrm{Precision}+\mathrm{Sensitivity}}$	(21)
$\;\mathrm{Accuracy}=\frac{\mathrm{TP}+\mathrm{TN}}{\mathrm{TP}+\mathrm{FP}+\mathrm{TN}+\mathrm{FN}}$	(22)		

**Table 5:** The genetic algorithm parameters settings for feature selection.

Parameter Name.	Types/Values
Population Size.	200
Genome Length	20
Selection type	Roulette wheel
Crossover	0.7 (Simple one-point crossover)
Mutation Probability	0.03 (Uniform probability distribution)
Maximum Generation No.	100

**Table 6:** The results of three classifiers in terms of six evaluation parameters.

	Precision	F1-Score	Sensitivity	Specificity	Accuracy (%)
**KNN**	0.97	0.97	0.989	0.988	97.0
**DT**	0.98	0.98	0.989	0.994	98.0
**RF**	0.99	0.99	0.994	0.997	99.7

**Table 7:** Comparing evaluation parameters of one-phase and two-phase current signal.

	KNN		DT		RF	
	2-phase signal	1-phase signal	2-phase signal	1-phase signal	2-phase signal	1-phase signal
Precision	0.97	0.90	0.98	0.91	0.99	0.91
F1-score	0.97	0.90	0.98	0.91	0.99	0.91
Sensitivity	0.989	0.86	0.989	0.91	0.994	0.92
Specificity	0.998	0.95	0.994	0.96	0.997	0.96
Accuracy (%)	97	89.70	98	91.03	99.7	91.12

**Table 8:** Accuracy comparison among the different methods.

Diagnosis Methods	Classification Accuracy (%)
MLP-based IF [52]	98.3
SVM-based IF [52]	98.0
KNN-based IF [52]	97.7
CNN with EWT [53]	97.37
CNN with generative oversampling (vibration signal) [54]	88.0–99.0
CNN (vibration signal) [55]	98.0–100
WPD + Ensemble learning [47]	86.03
**GA + KNN**	**97.0**
**GA + DT**	**98.0**
**GA + RF**	**99.7**
